# Supplementary material for: Altered receptor binding, antibody evasion and retention of T cell recognition by the SARS-CoV-2 XBB.1.5 spike protein
Source: Nat Commun. 2024 Feb 29;15:1854. doi: 10.1038/s41467-024-46104-2 (PMC10904792; doi:10.1038/s41467-024-46104-2)
Supplement: Supplementary file 1 — Supplementary Information [file 41467_2024_46104_MOESM1_ESM.pdf]

Supplementary Materials

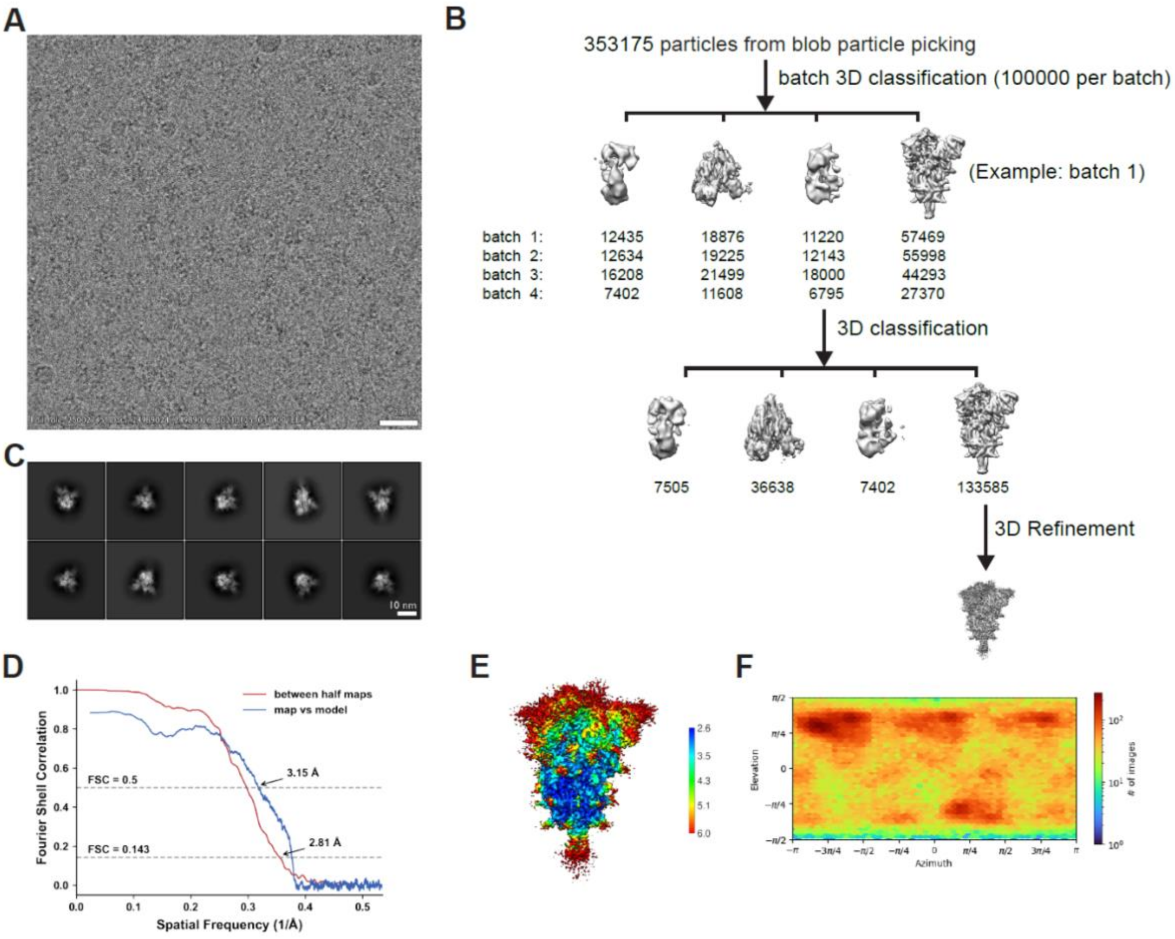

Supplementary Figure 1. Cryo-EM data processing and validation for the XBB.1.5 spike protein ectodomain. **(A)** Representative cryo-EM micrograph. **(B)** Workflow of cryo-EM image processing. **(C)** Representative 2D classes. **(D)** FSC curves. **(E)** Local resolution. **(F)** Viewing direction distribution plot.

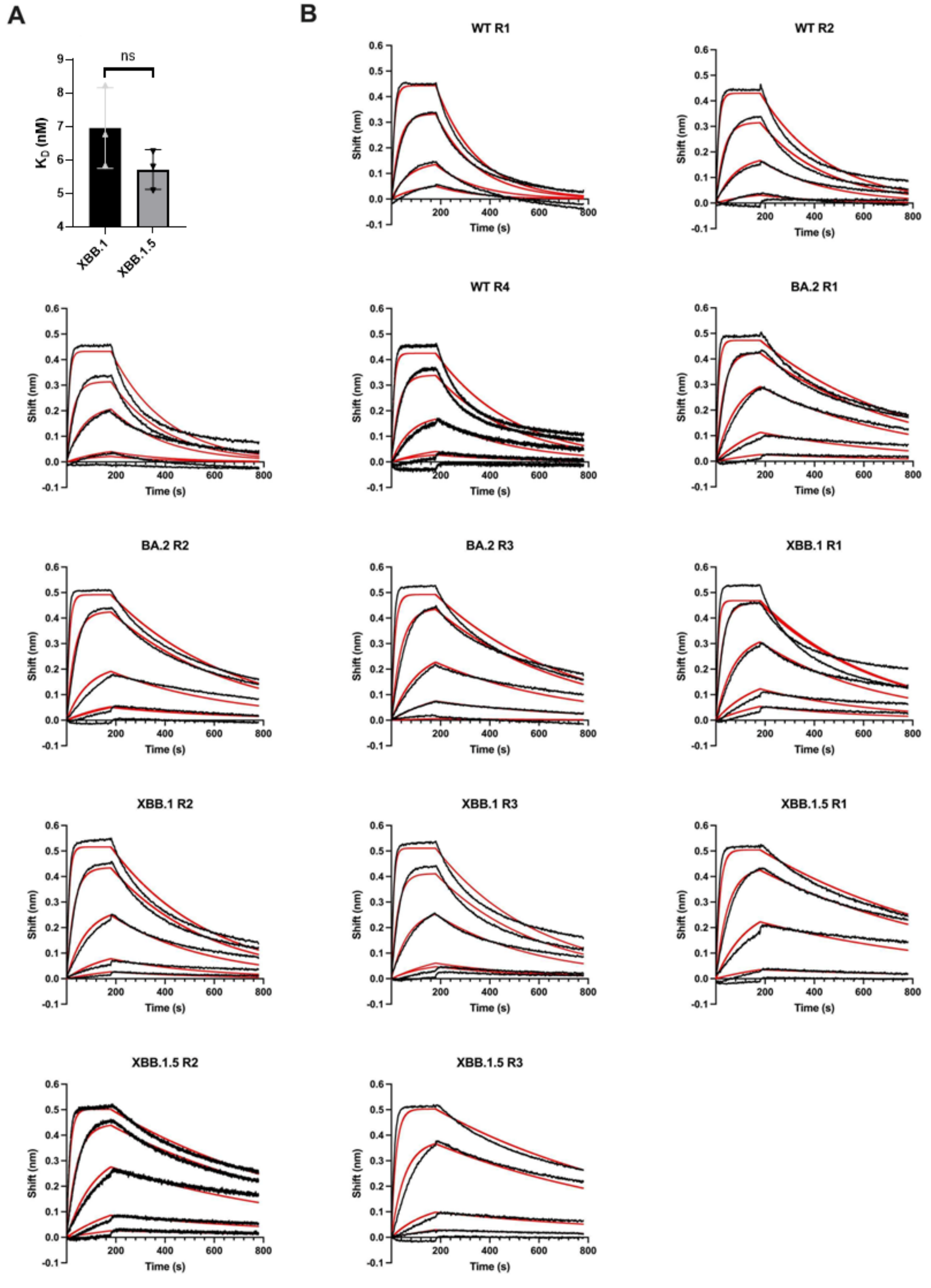

Supplementary Figure 2. BLI analysis of RBD – hACE2 interactions. (A) Summary of K_D values calculated for 3 experiments (n = 3) measuring XBB.1 and XBB.1.5 RBDs binding to hACE2. Error bars denote the standard deviation of the mean. Statistical significance was assessed via students T test (2-tailed, ns: not significant with alpha = 0.05) The calculated *P* value was 0.1856. (B) BLI curves for all RBD-hACE2 data in this study (R: replicate #). Black curves represent raw data which were fit to a model using a 1:1 binding stoichiometry (red) to determine the reported dissociation constants. Source data are provided as a Source Data file.

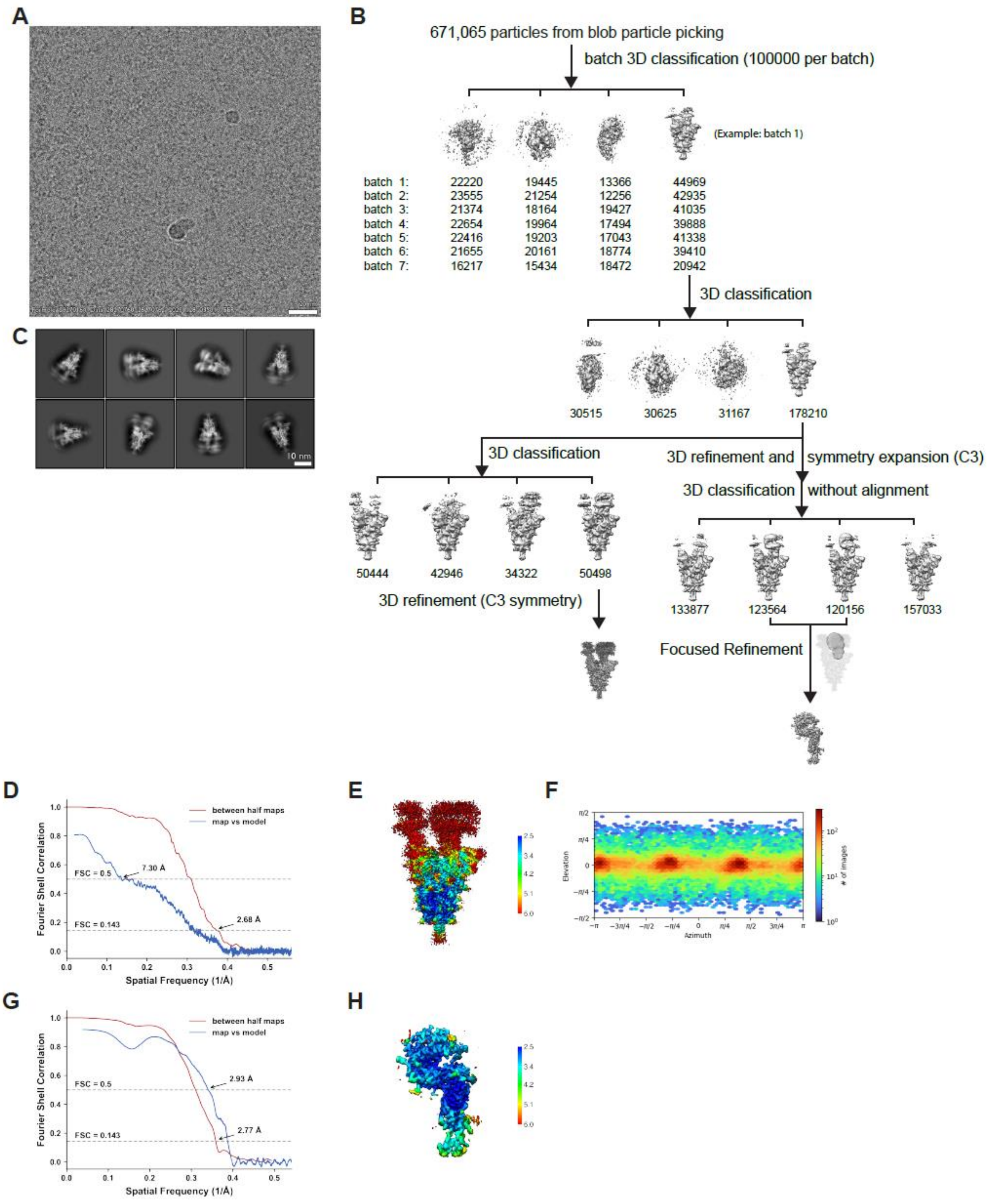

Supplementary Figure 3. Cryo-EM data processing and validation for complex of XBB.1.5 spike protein ectodomain and human ACE2. **(A)** Representative cryo-EM micrograph. **(B)** Workflow of cryo-EM image processing. **(C)** Representative 2D classes. **(D-F)** FSC curves **(D)**, local resolution **(E)** and viewing direction distribution plot **(F)** of global refinement. **(G-H)** FSC curves **(G)** and local resolution **(H)** of focused refinement.

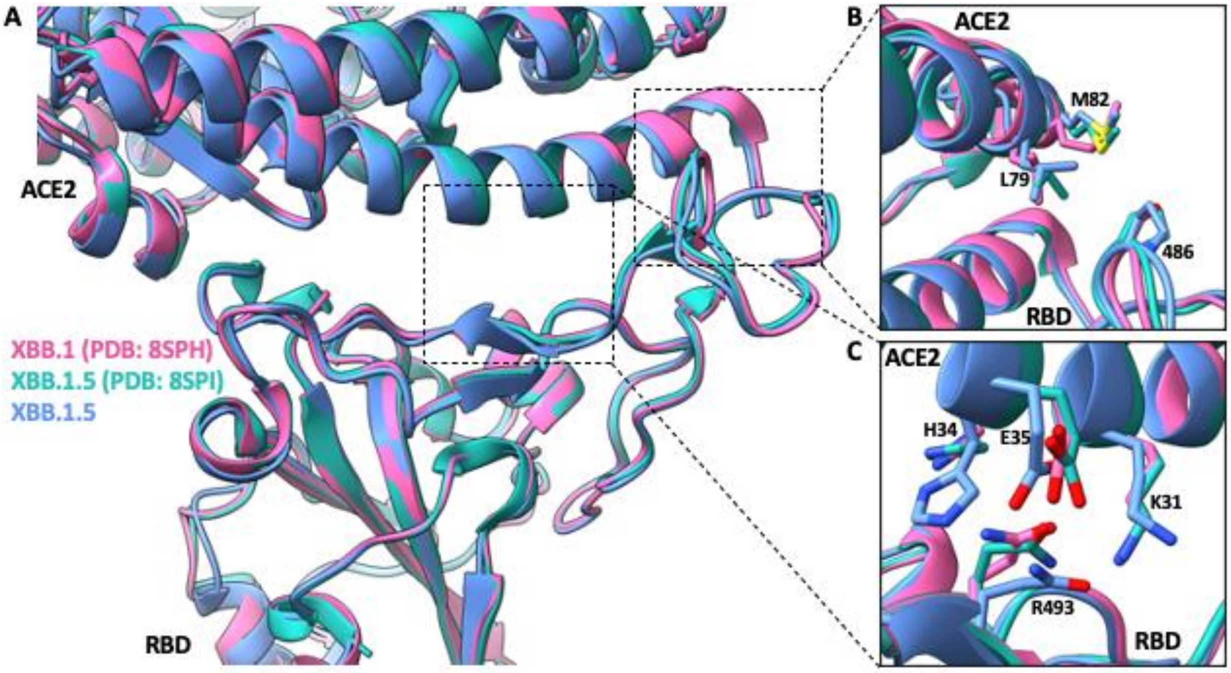

Supplementary Figure 4. Structural alignment between previously published chimeric XBB.1 and XBB.1.5 RBD-hACE2 complexes solved by X-ray crystallography. (A) XBB.1 (PDB ID: 8SPH), XBB.1.5 (PDB ID: 8SPI), and the focus-refined XBB.1.5 RBD - hACE2 cryoEM structure reported in this manuscript aligned by their RBDs. **(B)** Focused view on amino acid position 486 within the RBD and L79 and M82 residues within hACE2. **(C)** Focused view on residue 493 within the RBD and K31, H34, and E35 residues within hACE2.

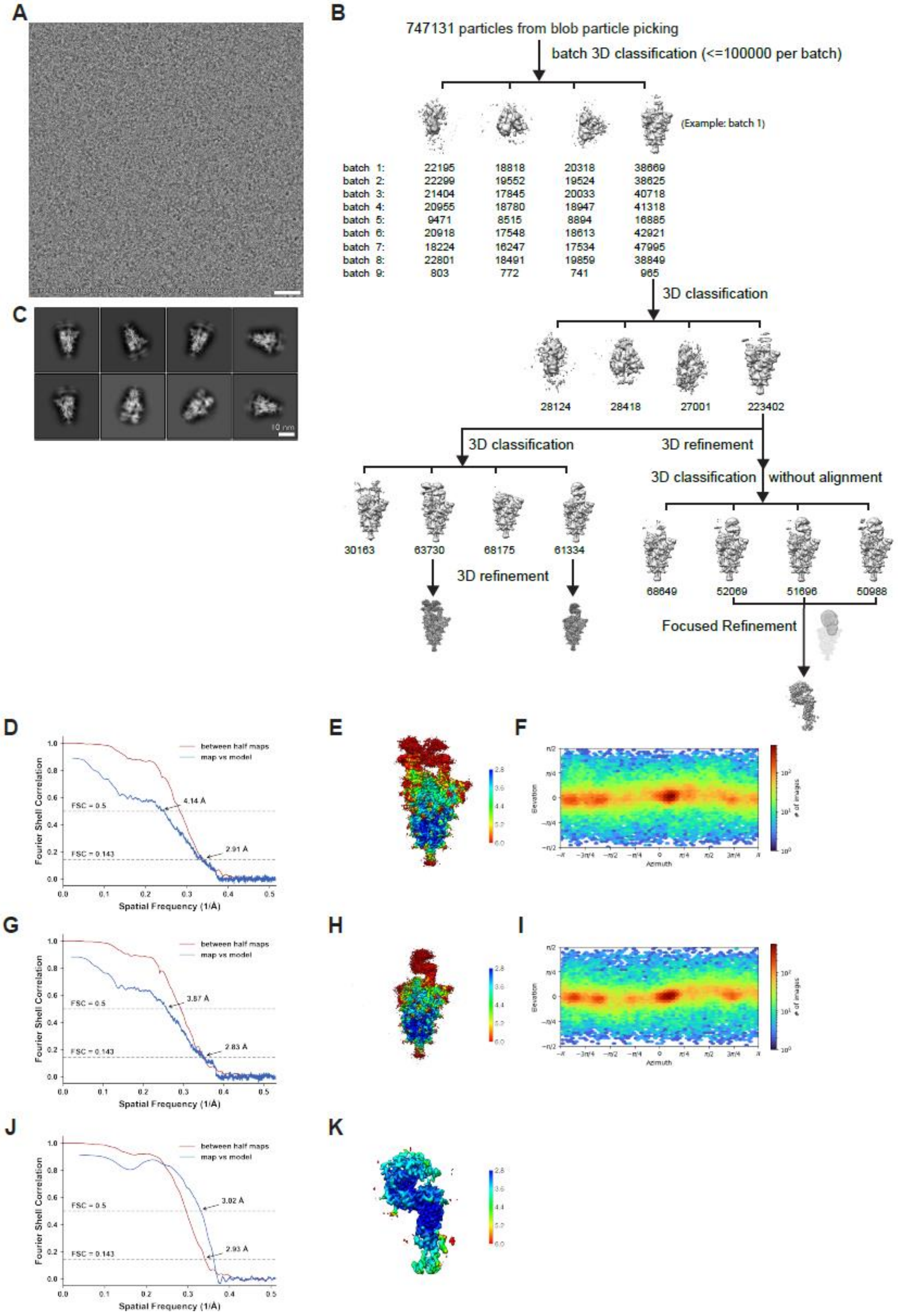

Supplementary Figure 5. Cryo-EM data processing and validation for complex of XBB.1.5 spike protein ectodomain and mouse ACE2. **(A)** Representative cryo-EM micrograph. **(B)** Workflow of cryo-EM image processing. **(C)** Representative 2D classes. **(D-F)** FSC curves **(D)**, local resolution **(E)** and viewing direction distribution plot **(F)** of two mouse ACE2 bound global refinement. **(G-I)** FSC curves **(G)**, local resolution **(H)** and viewing direction distribution plot **(I)** of one mouse ACE2 bound global refinement. **(J-K)** FSC curves **(J)**, and local resolution **(K)** of focused refinement.

A

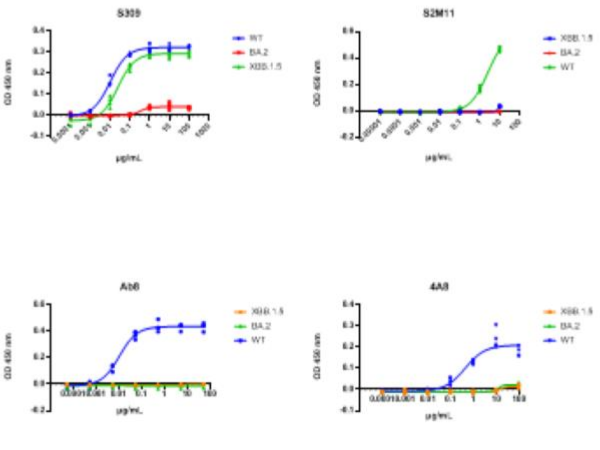
S309
WT
BA.2
XBB.1.5
OD 450 nm
µg/mL
S2M11
XBB.1.5
BA.2
WT
Ab8
XBB.1.5
BA.2
WT
4A8
XBB.1.5
BA.2
WT

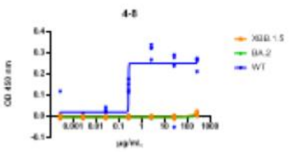
4-8
XBB.1.5
BA.2
WT
OD 450 nm
µg/mL

B

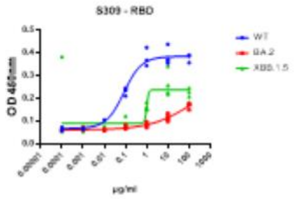
S309 - RBD
WT
BA.2
XBB.1.5
OD 450nm
µg/ml

C

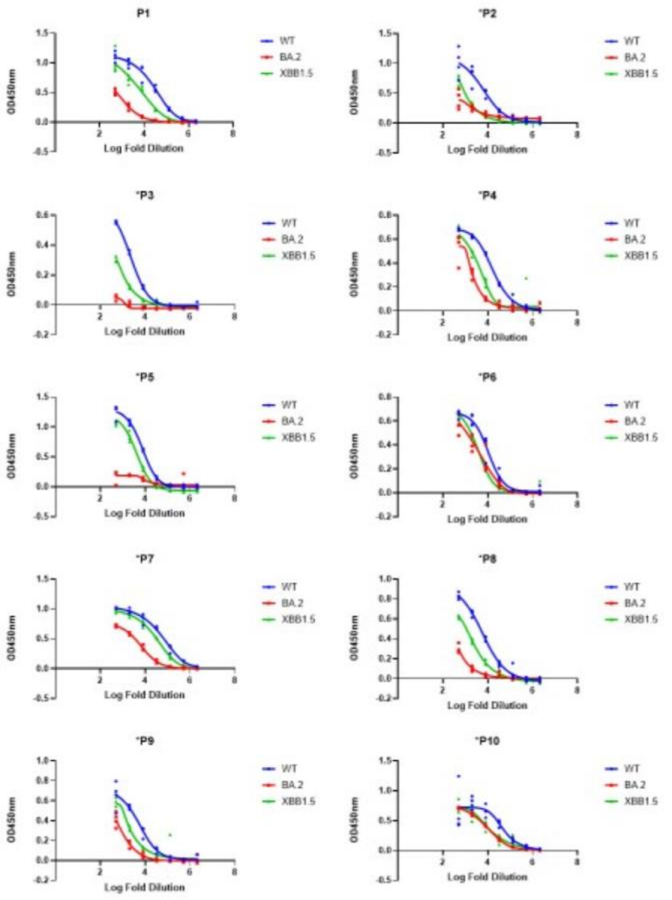
P1
*P2
*P3
*P4
*P5
*P6
*P7
*P8
*P9
*P10
WT
BA.2
XBB1.5
OD450nm
Log Fold Dilution

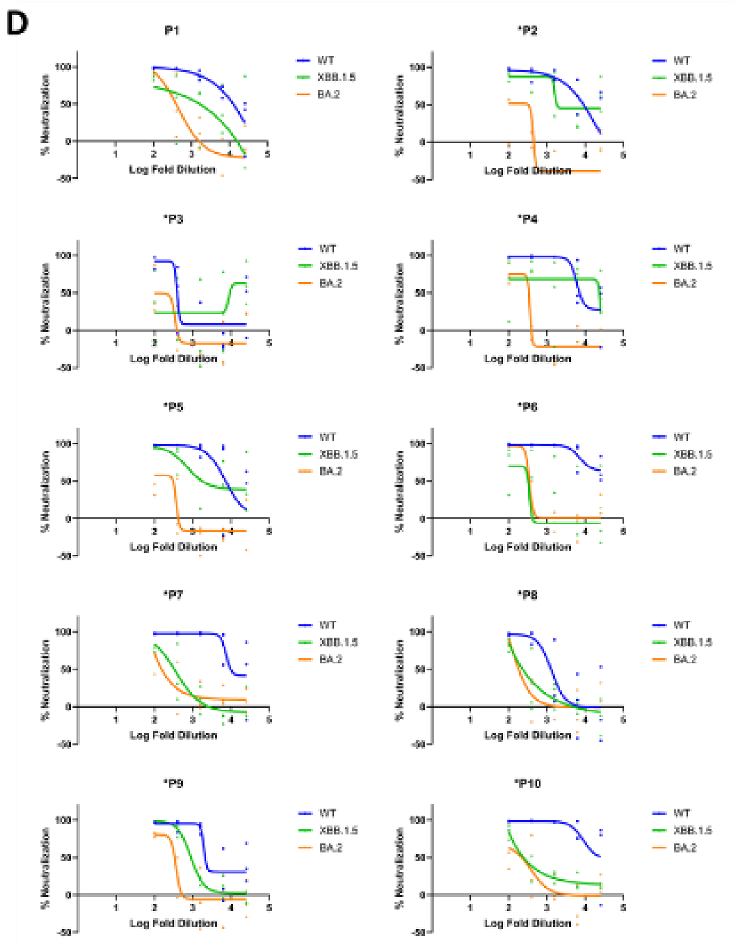

Supplementary Figure 6. ELISA and pseudovirus neutralization data. (A) Spike protein ELISAs for the indicated antibodies. **(B)** ELISA analysis of S309 binding the indicated spike protein RBDs. **(C)** Spike protein ELISAs using serum from donors. **(D)** Pseudoviral neutralization assays using serum from donors. All ELISA experiments were performed in technical quadruplicate and neutralization experiments in technical triplicate and are shown as points. Source data are provided as a Source Data file.

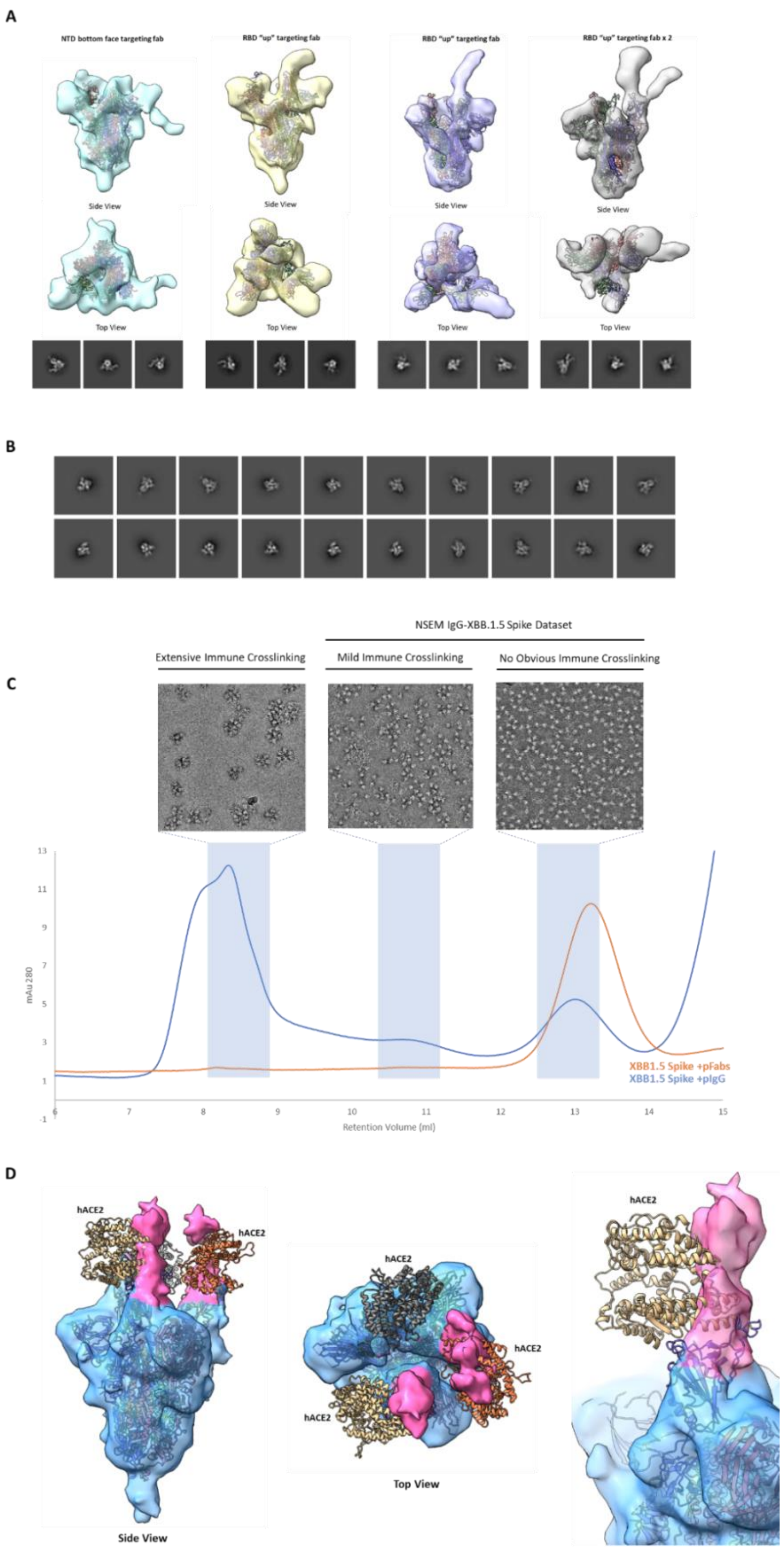

A
NTD bottom face targeting fab
RBD "up" targeting fab
RBD "up" targeting fab
RBD "up" targeting fab x 2
Side View
Top View
B
C
NSEM IgG-XBB.1.5 Spike Dataset
Extensive Immune Crosslinking
Mild Immune Crosslinking
No Obvious Immune Crosslinking
mAu 280
Retention Volume (ml)
XBB1.5 Spike +pFabs
XBB1.5 Spike +pIgG
D
hACE2
Side View
Top View

Supplementary Figure 7. Negative stain electron microscopy analysis of SARS-CoV-2 spike protein immune complexes. (A) Analysis of Fab-wild-type immune complexes. Side view and top views for each unique 3D reconstruction are shown, with representative 2D class averages below. Annotation of the antigenic regions recognized by Fab fragments in each reconstruction is provided at the top. Molecular models of spike proteins in various conformations are docked into each reconstruction (NTD bottom face reconstruction: open trimer with one RBD up and 2 down, PDB 6VYB, all others: open trimer with 3 RBDs up, PDB 7A98). **(B)** Representative 2D class averages of particles obtained when using bulk Fabs and the XBB.1.5 spike protein. **(C)** Analysis of IgG-XBB.1.5 spike protein complexes. A bulk IgG or bulk Fab-XBB.1.5 spike protein mixture was subjected to size exclusion chromatography. Traces for each injection are shown. Negative stain electron microscopy was performed on each indicated fraction from the IgG-XBB.1.5 spike protein mixture as indicated. **(D)** Fit of the XBB.1.5-hACE2 complex model into the obtained 3D reconstruction of an IgG-XBB.1.5 spike protein complex. Source data are provided as a Source Data file.

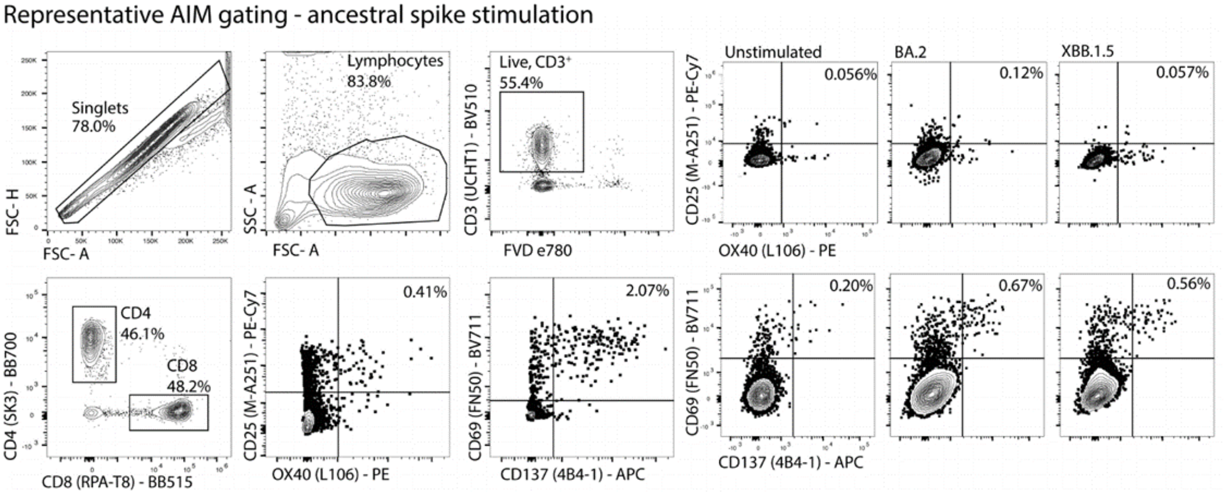

Supplementary Figure 8. Representative gating for T cell activation induced marker assay. FSC-H and FSC-A were used to gate single cells, followed by identification of lymphocytes using SSC-A and FSC-A. CD3 and Fixable Viability Dye (FVD) were used to gate for live T cells, which were further delineated by CD4 and CD8 expression. Antigen specific CD4$^+$ T cells were identified as CD25$^+$OX40$^+$ and CD8$^+$ antigen specific T cells as CD69$^+$CD137$^+$. Representative gating is shown for ancestral spike stimulated PBMCs, antigen specific gating is shown for all stimulation conditions.

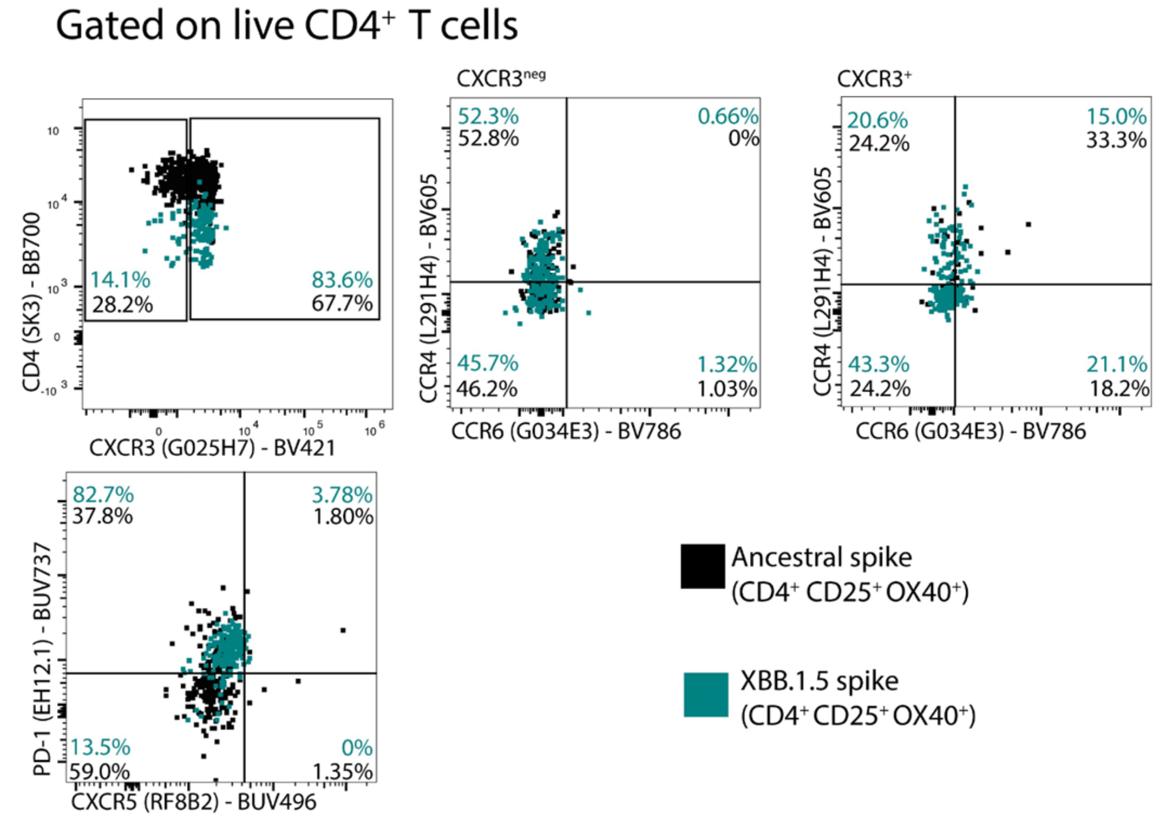

Supplementary Figure 9. Representative gating for antigen specific CD4$^+$ T cell phenotyping CD4$^+$ T cell phenotypic analysis was performed on samples with >30 CD25$^+$ OX40$^+$ T cells, which were analyzed for expression of CXCR3, followed by CCR4 and CCR6 expression. Th1 cells were identified as CXCR3$^+$ CCR4^{neg} CCR6^{neg}, Th2 cells as CXCR3^{neg} CCR4$^+$ CCR6^{neg}, Th9 cells as CXCR3$^+$ CCR4^{neg} CCR6$^+$, acute Th9 cells as CXCR3^{neg} CCR4^{neg} CCR6$^+$, Th17 cells as CXCR3^{neg} CCR4$^+$ CCR6$^+$, Th17.1 cells as CXCR3$^+$ CCR4$^+$ CCR6$^+$. Circulating CD4$^+$ Tfh (cTfh) cells were defined as being CXCR5$^+$ and PD-1$^+$.

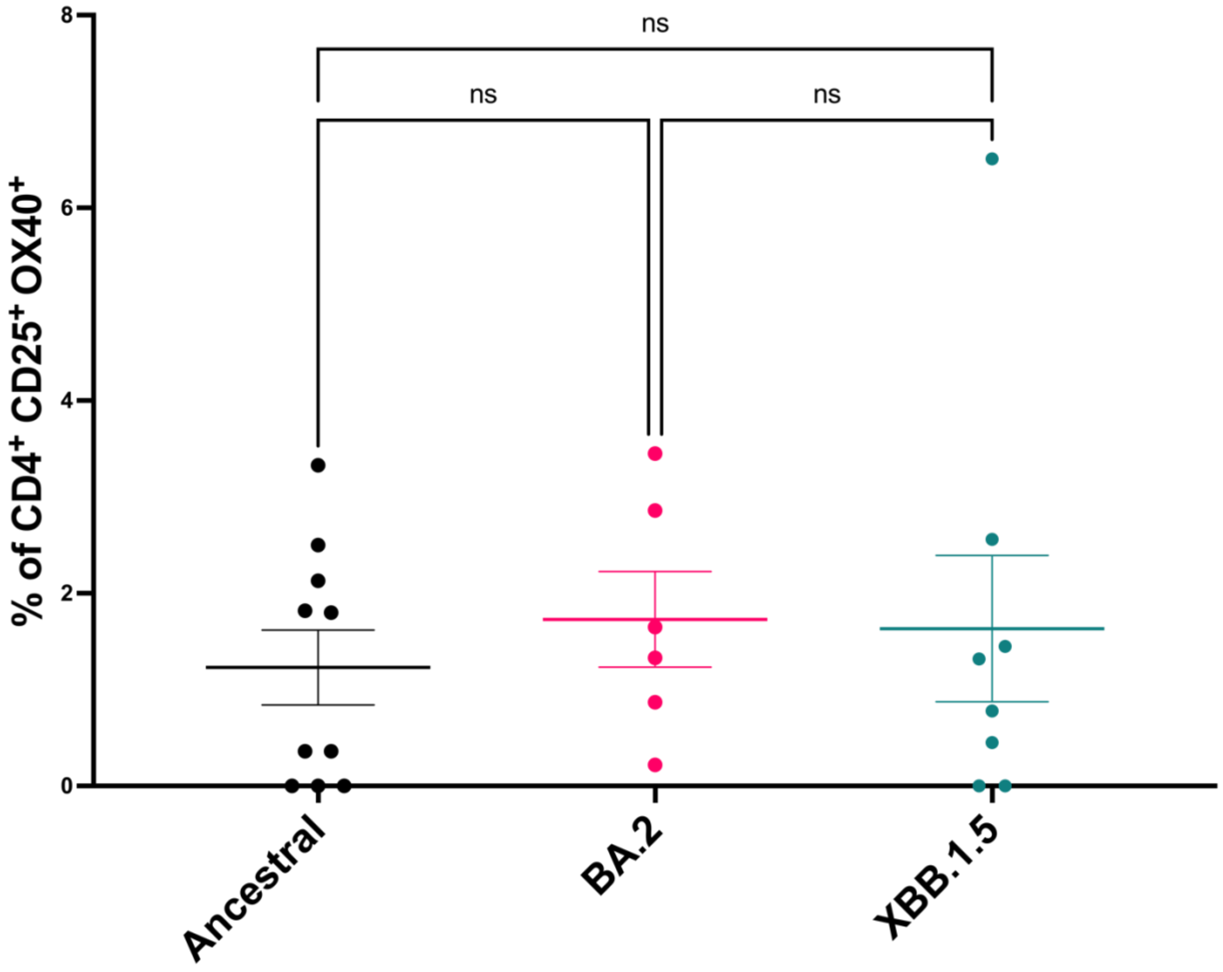

Supplementary Figure 10. Spike specific circulating T follicular helper (cTfh) cells $CD4^+$ T cell phenotypic analysis was performed on samples with > 30 $CD25^+$ $OX40^+$ T cells, with cTfh cells identified by surface expression of CXCR5 and PD-1. Black, Magenta, Teal points represent data for the Ancestral (wild type/WT), BA.2, and XBB.1.5 spike proteins respectively; ns: not significant. Kruskal-Wallis tests with Dunn's multiple comparisons tests were used to determine significant differences. Calculated P values are as follows: WT vs BA.2: $P > 0.9999$, WT vs XBB.1.5: $P > 0.9999$, BA.2 vs XBB.1.5: $P = 0.6826$. Source data are provided as a Source Data file.

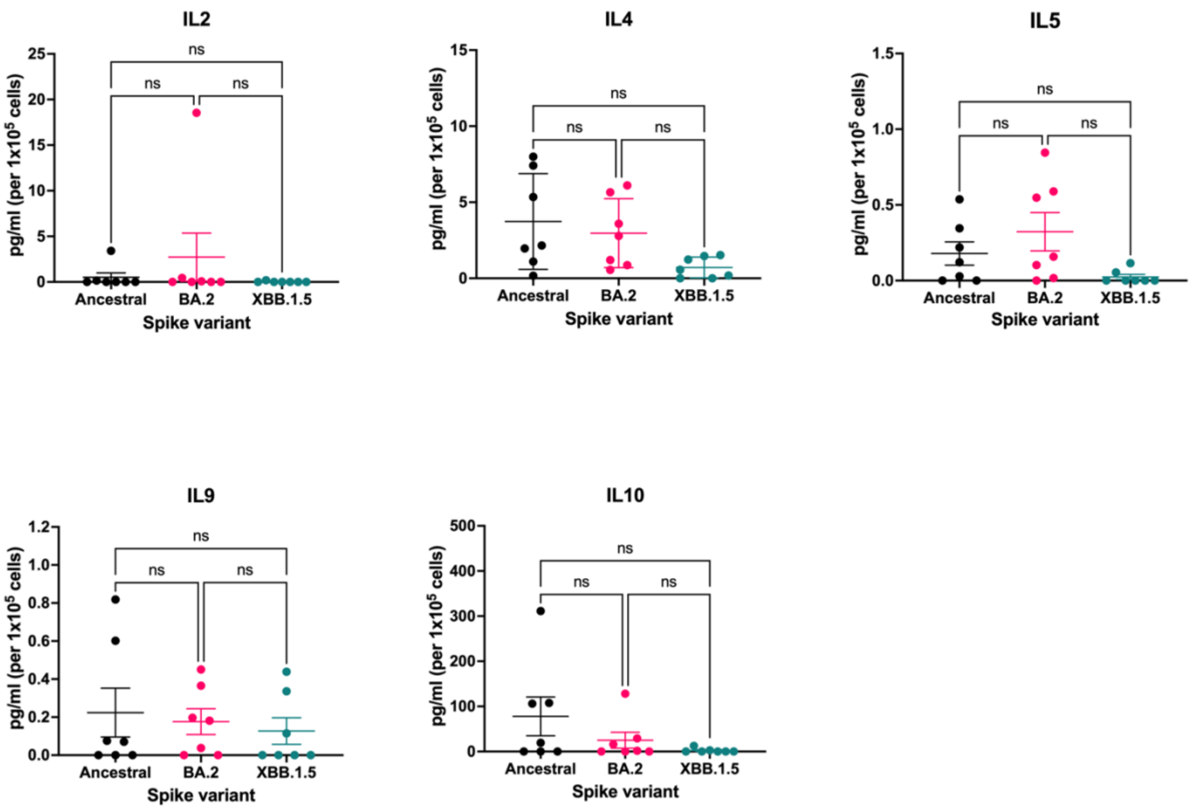

Supplementary Figure 11. Cytokine levels following stimulation of PBMCs with spike proteins Cell culture supernatants from 44-hour spike stimulated PBMCs were analyzed using a LEGENDPlex assay. Unstimulated cytokine levels were subtracted from each condition and concentrations (pg/ml) were normalized to 10^5 cells. Black, Magenta, Teal points represent data for the Ancestral (wild type/WT), BA.2, and XBB.1.5 spike proteins respectively; ns: not significant. Kruskal-Wallis tests with Dunn's multiple comparisons tests were used to determine significant differences. Calculated P values are as follows: (IL2: P = 0.4905), (IL4: P = 0.0.0602), (IL5: WT vs BA.2 (P > 0.9999), WT vs XBB.1.5 (P = 0.2900), BA.2 vs XBB.1.5 (P = 0.0.0503), (IL9: P = 0.7132), (IL10: P = 0.2571). Source data are provided as a Source Data file.

Supplementary Table 1. Data collection and processing parameters, refinement, and validation statistics.

Structure:	S(XBB.1.5)	S(XBB.1.5) + hACE2		S(XBB.1.5) + mACE2		
	global refinement	global refinement	focus refinement	global refinement 1	global refinement 2	focus refinement
	EMD-43320	EMD-43324	EMD-43325	EMD-43321	EMD-43322	EMD-43323
	PDB ID: 8VKK	PDB ID: 8VKO	PDB ID: 8VKP	PDB ID: 8VKL	PDB ID: 8VKM	PDB ID: 8VKN
**Data collection**						
Microscope	Titan Krios G4	Titan Krios G4		Titan Krios G4		
Detector	Falcon4	Falcon4		Falcon4		
Voltage (kV)	300	300		300		
Nominal magnification	155,000	155,000		155,000		
Defocus range (μm)	-2.0 to -0.5	-2.0 to -0.5		-2.0 to -0.5		
Physical pixel (Å)	0.5	0.5		0.5		
Electron dose ($e^-/Å^2$)	40	40		40		
Exposure rate ($e^-/Å^2$/sec)	24	24		24		
Format of movies	EER	EER		EER		
Number of raw frames	413	413		413		
Number of movies	4,185	8,064		9,152		
**Data processing**						
Number of fractions	40	40		40		
Number of extracted particles	353,175	671,065		747,131		
Number of particles for final map	133,585	50,498	243720*	63,730	61,334	154,753
Symmetry imposed	C1	C3	C1	C1	C1	C1
Resolution (Å)	2.81	2.68	2.77	2.91	2.83	2.93
FSC threshold	0.143	0.143	0.143	0.143	0.143	0.143
**Refinement**						
Initial model used	8DM1	8DM1,8DM6	8DM6	8DM1,8DM8	8DM1,8DM8	8DM8
Map sharpening B-factor ($Å^2$)	47.6	40.7	72.8	34.8	34.6	72.0
Composition (#)						
Atoms	23,808	40,293	6,577	35,186	30,286	6,508
Residues	2,944	4,926	796	4,327	3,734	794
Ligands	NAG:56	NAG:81	NAG:9	BMA:2; NAG:65	BMA:1; NAG:61	BMA:1; NAG:5
B-factor ($Å^2$)						
Protein	123.88	111.95	101.84	111.12	110.33	110.86
Ligand	123.33	100.91	111.02	112.85	116.73	129.64
Bonds (RMSD)						
Length (Å) (# > 4σ)	0.009 (0)	0.005 (0)	0.007 (0)	0.005 (0)	0.005 (0)	0.006 (0)
Angles (°) (# > 4σ)	0.948 (9)	0.883 (9)	0.997 (1)	0.898 (8)	0.875 (6)	0.933 (0)
CC_mask	0.80	0.46	0.82	0.58	0.62	0.84
**Validation**						
Ramachandran plot						
Residues favored (%)	98.06	97.41	96.84	98.36	97.69	96.84
Residues disallowed (%)	0.00	0.00	0.00	0.00	0.00	0.00
Rotamer outliers (%)	0.58	1.06	1.14	1.11	0.95	0.87
Clash score	3.64	3.16	3.35	3.39	3.67	3.46
MolProbity score	1.15	1.24	1.36	1.16	1.22	1.33

* particles are derived from symmetry expansion and 3D classification without alignment.

Supplementary Table 2. Donor demographics for serum and PMBC studies. The average age of donors is 31.7 years.

ID	Sex	Vaccine type	Number of doses	Bivalent	Previous Infection	Infection date estimate
1	m	AZ (ChAdOx1-S) + 4 Moderna (mRNA-1273)	5	Y	Y	n/a
2	f	Covax	2	N	Y	May-21
3	f	Pfizer (BNT162b2)	3	n/a	Y	n/a
4	m	Pfizer (BNT162b2 x 3 + Moderna (mRNA-1273)	4	Y	Y	Mar-22
5	f	Pfizer (BNT162b2)	4	Y	Y	Jan-22
6	f	Pfizer (BNT162b2)	4	Y	Y	n/a
7	f	Pfizer (BNT162b2) x2 Moderna (mRNA-1273) x1	3	N	Y	Aug-22
8	m	Moderna (mRNA-1273)	2	N	Y	ancestral
9	m	Pfizer (BNT162b2)	4	Y	Y	Jan-22
10	f	Moderna (mRNA-1273)/Pfizer (BNT162b2)	3	Y	Y	Jan-22

Supplementary Table 3. mAb panel for T cell assays

Target	Fluorochrome	Clone	Company	Dilution
CXCR5	BUV496	RF8B2	BD Biosciences	1:100
PD-1	BUV737	EH12.1	BD Biosciences	1:100
CXCR3	BV421	G025H7	Biolegend	1:100
CD3	BV510	UCHT1	BD Biosciences	1:200
CCR4	BV605	L291H4	Biolegend	1:200
CD69	BV711	FN50	Biolegend	1:100
CCR6	BV786	G034E3	Biolegend	1:100
CD8	BB515	PA-T8	BD Biosciences	1:200
CD4	BB700	SK3	BD Biosciences	1:200
OX40	PE	L106	BD Biosciences	1:10
CD39	PE-Dazzle594	A1	Biolegend	1:100
CD25	PE-Cy7	M-A251	BD Biosciences	1:40
CD137	APC	4B4-1	Biolegend	1:100
FVD	eFluor 780	NA	ThermoFisher	1:1000
