# Supplementary material for: Altered receptor binding, antibody evasion and retention of T cell recognition by the SARS-CoV-2 XBB.1.5 spike protein
Source: Nat Commun. 2024 Feb 29;15:1854. doi: 10.1038/s41467-024-46104-2 (PMC10904792; doi:10.1038/s41467-024-46104-2)
Supplement: Supplementary file 3 — Reporting Summary [file 41467_2024_46104_MOESM3_ESM.pdf]

nature portfolio

Corresponding author(s): Dr. Sriram Subramaniam

Last updated by author(s): Jan 10, 2024

Reporting Summary

Nature Portfolio wishes to improve the reproducibility of the work that we publish. This form provides structure for consistency and transparency in reporting. For further information on Nature Portfolio policies, see our Editorial Policies and the Editorial Policy Checklist.

Statistics

For all statistical analyses, confirm that the following items are present in the figure legend, table legend, main text, or Methods section.

n/a	Confirmed	
x		The exact sample size (n) for each experimental group/condition, given as a discrete number and unit of measurement
	x	A statement on whether measurements were taken from distinct samples or whether the same sample was measured repeatedly
	x	The statistical test(s) used AND whether they are one- or two-sided<br>*Only common tests should be described solely by name; describe more complex techniques in the Methods section.*
x		A description of all covariates tested
x		A description of any assumptions or corrections, such as tests of normality and adjustment for multiple comparisons
	x	A full description of the statistical parameters including central tendency (e.g. means) or other basic estimates (e.g. regression coefficient) AND variation (e.g. standard deviation) or associated estimates of uncertainty (e.g. confidence intervals)
	x	For null hypothesis testing, the test statistic (e.g. F, t, r) with confidence intervals, effect sizes, degrees of freedom and P value noted<br>*Give P values as exact values whenever suitable.*
x		For Bayesian analysis, information on the choice of priors and Markov chain Monte Carlo settings
x		For hierarchical and complex designs, identification of the appropriate level for tests and full reporting of outcomes
x		Estimates of effect sizes (e.g. Cohen's d, Pearson's r), indicating how they were calculated

Our web collection on statistics for biologists contains articles on many of the points above.

Software and code

Policy information about availability of computer code

Data collection	EPU2 automated acquisition (Thermo Fisher Scientific; https://www.thermofisher.com/us/en/home/electron-microscopy/products/software-em-3d-vis/epu-software.html)
Data analysis	GraphPad Prism (GraphPad 9.0; https://www.graphpad.com/scientific-software/prism/)<br>UCSF Chimera (v.1.16)(Eric F. Pettersen TDG, Conrad C. Huang, Gregory S. Couch, Daniel M. Greenblatt, Elaine C. Meng, Thomas E. Ferrin. UCSF Chimera—A visualization system for exploratory research and analysis. Journal of Computational Chemistry 25, 1605-1612 (2004). https://www.cgl.ucsf.edu/chimera/)<br>crYOLO (version 1.7.4) (Wagner T, et al. SPHIRE-crYOLO is a fast and accurate fully automated particle picker for cryo-EM. Communications Biology 2, (2019). https://pypi.org/project/cryolo/)<br>cryoSPARC live (v3.0.1) (Punjani A, Rubinstein JL, Fleet DJ, Brubaker MA. cryoSPARC: algorithms for rapid unsupervised cryo-EM structure determination. Nature Methods 14, 290-296 (2017). https://cryosparc.com/live)<br>MolProbity (v4.5.l) (Chen, V. B. et al. MolProbity: all-atom structure validation for macromolecular crystallography. Acta Crystallogr. D. Biol. Crystallogr. 66, 12-21 (2010). http://molprobity.biochem.duke.edu/)

For manuscripts utilizing custom algorithms or software that are central to the research but not yet described in published literature, software must be made available to editors and reviewers. We strongly encourage code deposition in a community repository (e.g. GitHub). See the Nature Portfolio guidelines for submitting code & software for further information.

Data

Policy information about availability of data

All manuscripts must include a data availability statement. This statement should provide the following information, where applicable:

- Accession codes, unique identifiers, or web links for publicly available datasets
- A description of any restrictions on data availability
- For clinical datasets or third party data, please ensure that the statement adheres to our policy

The atomic models and cryo-EM density maps have been deposited into the Protein Data Bank (PDB) and Electron Microscopy Data Bank (EMDB) as follows:
XBB.1.5 spike protein (PDB ID: 8VKK, EMDB ID: EMD-43320)
mACE2 x2 + XBB.1.5 S (PDB ID: 8VKL, EMDB ID: EMD-43321)
mACE2 + XBB.1.5 S (PDB ID: 8VKM EMDB ID: EMD-43322)
Focused refined mACE2 + XBB1.5 RBD (PDB ID: 8VKN, EMDB ID: EMD-43323)
hACE2x3 + XBB.1.5 S (PDB ID: 8VKO, EMDB ID: EMD-43324)
Focused refined hACE2 + XBB1.5 RBD (PDB ID: 8VKP, EMDB ID: EMD-43325)
Negative Stain EM Reconstructions of SARS-CoV-2 spike proteins mixed with polyclonal antibodies from donor 4 (EMDB ID: EMD-43325)

Research involving human participants, their data, or biological material

Policy information about studies with human participants or human data. See also policy information about sex, gender (identity/presentation), and sexual orientation and race, ethnicity and racism.

Reporting on sex and gender	Sex was determined based on self-reporting and was not considered in the study design. Four male participants and six female participants were included in this study as summarized in Supplementary Table S2.
Reporting on race, ethnicity, or other socially relevant groupings	No reporting on race, ethnicity, or other socially relevant grouping was conducted.
Population characteristics	Sex, age, and vaccination status were the three population characteristic information obtained from participants (as summarized in Supplementary Table S2).
Recruitment	Participants were volunteers in research laboratories at BC Childrens Hospital and the Djavad Mowafaghian Centre for Brain Health. Population characteristics are summarized in Supplementary Table S2.
Ethics oversight	COVID-19 Immunity Study Samples were collected by Dr. Ted Steiner, with UBC ethic approval (UBC Clinical Research Ethics Board), certificate number: H20-00966.

Note that full information on the approval of the study protocol must also be provided in the manuscript.

Field-specific reporting

Please select the one below that is the best fit for your research. If you are not sure, read the appropriate sections before making your selection.

[x] Life sciences [ ] Behavioural & social sciences [ ] Ecological, evolutionary & environmental sciences

For a reference copy of the document with all sections, see nature.com/documents/nr-reporting-summary-flat.pdf

Life sciences study design

All studies must disclose on these points even when the disclosure is negative.

Sample size	Patient-derived serum psuedovirus neutralization studies<br>-Sample size was not predetermined and the sample size presented in the manuscript reflects the availability of vaccine-induced and convalescent patient derived serum samples.<br>Patient-derived T-cellular (AIM) study<br>-Sample size was not predetermined and the sample size presented in the manuscript reflects the availability of vaccine-induced and convalescent patient derived serum samples.
Data exclusions	No data was excluded from this study
Replication	Patient-derived serum psuedovirus neutralization studies<br>-Experiments were performed in technical triplicate. All replication attempts were successful.<br>Patient-derived T-cellular (AIM) study<br>-Experiments were performed in individual measurements with at least 100,000 events acquired for each condition.<br>Surface plasmon resonance study<br>-Experiments were conducted in single measurements due to limited instrument access. Results were corroborated by biolayer interferometry studies which were performed in technical triplicate measurements as stated below.

Biolayer interferometry study
-Experiments were performed in technical triplicate measurements to conform with field-specific conventions. Results were corroborated by surface plasmon resonance studies as stated above. All replication attempts were successful.
ELISA mACE2 binding study
-Experiments were performed in technical triplicates. All replication attempts were successful.
ELISA monoclonal antibody binding study
-Experiments were performed in technical triplicate measurements. All replication attempts were successful.
ELISA polyclonal antibody binding study
-Experiments were performed in technical quadruplicate. All replication attempts were successful.

Randomization

Our samples were not randomized as all samples were treated identically within the same experimental design.

Blinding

Blinding was not relevant to this study as all measurements were obtained by quantitative biochemical assays and not through subjective means.

Reporting for specific materials, systems and methods

We require information from authors about some types of materials, experimental systems and methods used in many studies. Here, indicate whether each material, system or method listed is relevant to your study. If you are not sure if a list item applies to your research, read the appropriate section before selecting a response.

Materials & experimental systems

n/a	Involved in the study
☐	☒ Antibodies
☐	☒ Eukaryotic cell lines
☒	☐ Palaeontology and archaeology
☒	☐ Animals and other organisms
☒	☐ Clinical data
☒	☐ Dual use research of concern
☒	☐ Plants

Methods

n/a	Involved in the study
☒	☐ ChIP-seq
☐	☒ Flow cytometry
☒	☐ MRI-based neuroimaging

Antibodies

Antibodies used

VH ab8 (Li W, et al. PNAS 2020; Li W, et al Cell 2020)
Fab S309 (Pinto D, et al. Nature 2020)
Fab S2M11 (Tortirici MA, et al. Science 2020)
Fab 4A8 (Chi X, et al. Science 2020)
Fab 4-8 (Liu L, et al. Nature 2020)
Goat anti-human IgG - HRP (Jackson ImmunoResearch, Cat. # 109-035-088)
IgG (H+L) Cross-Adsorbed Goat anti-Rabbit, DyLight 680, (Invitrogen, Cat. # PI35569)
Goat anti-Mouse IgG (H+L) Cross-Adsorbed Secondary Antibody, Alexa Fluor 647, (Invitrogen, Cat. # A-21235)
Anti-Strep-Tag-Antibody (BIO_RAD, Cat. # MCA2489)

Validation

The validation of each primary antibody is described in their associated publication, as referenced in the above section.
Validation documentation for the Goat anti-human IgG - HRP antibody can be found at this webpage: https://www.jacksonimmuno.com/catalog/products/109-035-088
IgG (H+L) Cross-Adsorbed Goat anti-Rabbit: https://www.fishersci.com/shop/products/goat-anti-rabbit-igg-h-l-dylight-680-polyclonal-thermoscientific-pierce-dylight-680/PI135569
Goat anti-Mouse IgG (H+L) Cross-Adsorbed Secondary Antibody: https://www.thermofisher.com/antibody/product/Goat-anti-Mouse-IgG-H-L-Cross-Absorbed-Secondary-Antibody-Polyclonal/A-21235
Anti-Strep-Tag-Antibody: https://www.bio-rad-antibodies.com/monoclonal/synthetic-peptide-strep-tag-classicantibody-strep-tag-ii-mca2489.html?f=purified

Eukaryotic cell lines

Policy information about cell lines and Sex and Gender in Research

Cell line source(s)

Expi293F (Thermo Fisher Scientific; cat# A14527)
HEK293T-ACE2-TMPRSS2 cells (BEI Resources; cat# NR-55293)

Authentication

Expi293F - please see this webpage for authentication documentation: https://www.thermofisher.com/order/catalog/product/A14527#/A14527

HEK293T-ACE2-TMPRSS2 cells - please see this webpage for authentication documentation: https://www.beiresources.org/Catalog/cellBanks/NR-55293.aspx

Mycoplasma contamination	Cell lines were not tested for mycoplasma contamination, as they were used solely for protein expression and pseudovirus neutralization assays.
Commonly misidentified lines (See ICLAC register)	N/A

Flow Cytometry

Plots

Confirm that:

- [x] The axis labels state the marker and fluorochrome used (e.g. CD4-FITC).
- [ ] The axis scales are clearly visible. Include numbers along axes only for bottom left plot of group (a 'group' is an analysis of identical markers).
- [x] All plots are contour plots with outliers or pseudocolor plots.
- [x] A numerical value for number of cells or percentage (with statistics) is provided.

Methodology

Sample preparation	Whole heparinized blood was obtained from healthy donors via venipuncture. PBMCs were isolated by centrifugation of blood over Ficoll and stored in liquid nitrogen. Prior to stimulation, PBMCs were thawed and rested overnight in Immunocult media (StemCell Technologies) at 0.5-1 x 106 cells/mL in a 96-well plate. Cells were stimulated with 2 µg/ml of ancestral, BA.2, or XBB.1.5 spike protein or left unstimulated. Equal volumes of protein buffer (20mM Tris pH8 150mM NaCl) were added to each unstimulated condition. 2µL of anti-CD137-APC mAb was added to each well at the time of stimulation. PBMCs were incubated at 37C for 44-hours. Following stimulation, cells were centrifuged, and supernatant was collected and stored at -80C. Pellets were washed and resuspended in PBS and stained for 20 minutes with a cocktail of the antibodies in Supplementary Table 2. Following staining, cells were then washed twice with PBS, resuspended in 0.5% paraformaldehyde in PBS, and acquired on a 5-laser Symphony flow cytometer (BD Biosciences). A minimum of 100,000 events were acquired for each condition. Flow cytometry data analysis was performed using FlowJo v10.8.1 (BD Biosciences).
Instrument	5-laser Symphony flow cytometer (BD Biosciences)
Software	FlowJo v10.8.1 (BD Biosciences)
Cell population abundance	A minimum of 100,000 events were acquired for each condition.
Gating strategy	Please see supplemental figure S8.

- [x] Tick this box to confirm that a figure exemplifying the gating strategy is provided in the Supplementary Information.
